# Challenges in institutionalizing evidence-informed priority setting for health service packages: a qualitative document and interview analysis from Iran

**DOI:** 10.1186/s12961-024-01207-6

**Published:** 2024-08-19

**Authors:** Haniye Sadat Sajadi, Hamidreza Safikhani, Alireza Olyaeemanesh, Reza Majdzadeh

**Affiliations:** 1 Knowledge Utilization Research Center, University Research and Development Center, Tehran University of Medical Sciences, Tehran, Iran; 2 Iranian Health Economics Association, Tehran, Iran; 3 National Institute of Health Research and Health Equity Research Center, Tehran University of Medical Sciences, Tehran, Iran; 4 School of Health and Social Care, University of Essex, Colchester, United Kingdom

**Keywords:** Benefits package, Evidence-based practice, Health policy, Health financing, Knowledge translation, Universal health coverage

## Abstract

**Background:**

Setting and implementing evidence-informed health service packages (HSPs) is crucial for improving health and demonstrating the effective use of evidence in real-world settings. Despite extensive training for large groups on evidence generation and utilization and establishing structures such as evidence-generation entities in many countries, the institutionalization of setting and implementing evidence-informed HSPs remains unachieved. This study aims to review the actions taken to set the HSP in Iran and to identify the challenges of institutionalizing the evidence-informed priority-setting process.

**Methods:**

Relevant documents were obtained through website search, Google queries, expert consultations and library manual search. Subsequently, we conducted nine qualitative semi-structured interviews with stakeholders. The participants were purposively sampled to represent diverse backgrounds relevant to health policymaking and financing. These interviews were meticulously audio-recorded, transcribed and reviewed. We employed the framework analysis approach, guided by the Kuchenmüller et al. framework, to interpret data.

**Results:**

Efforts to incorporate evidence-informed process in setting HSP in Iran began in the 1970s in the pilot project of primary health care. These initiatives continued through the Health Transformation Plan in 2015 and targeted disease-specific efforts in 2019 in recent years. However, full institutionalization remains a challenge. The principal challenges encompass legal gaps, methodological diversity, fragile partnerships, leadership changeovers, inadequate financial backing of HSP and the dearth of an accountability culture. These factors impede the seamless integration and enduring sustainability of evidence-informed practices, hindering collaborative decision-making and optimal resource allocation.

**Conclusions:**

Technical aspects of using evidence for policymaking alone will not ensure sustainability unless it achieves the necessary requirements for institutionalization. While addressing all challenges is crucial, the primary focus should be on required transparency and accountability, public participation with an intersectionality lens and making this process resilience to shocks. It is imperative to establish a robust legal framework and a strong and sustainable political commitment to embrace and drive change, ensuring sustainable progress.

## Background

Universal health coverage (UHC) is a global commitment to effective coverage of needed services and ensuring no one left behind them, a target all nations have pledged to achieve certain targets by 2030. However, the most recent annual reports on UHC monitoring paint a disheartening picture [1]. Countries still have a considerable distance to reach UHC targets for 2030, raising concerns about whether health resources are sufficiently allocated to attain the UHC.

One of the fundamental aspects of UHC is service coverage. By its very definition, UHC explicitly emphasizes providing needed services with adequate quality to everyone without financial hardship [2]. Therefore, one of the primary strategies for moving towards UHC is to understand the list of needed services through setting a health service package (HSP) considering country’s financial realities and social preferences [3].

Experiences across various nations suggest that many are striving to set HSP. However, health needs are evolving, technologies and services are advancing and financial resources are also undergoing changes. It demands ongoing updates to the HSP. Consequently, the institutionalization of its setting and implementation is of significant importance. Recent reviews of countries’ endeavours in setting HSP also highlight that institutionalization poses a challenge for most nations [4, 5].

The health system in Iran underwent significant transformation after the Islamic Revolution of 1979. This revolution, which led to the overthrowing of the Pahlavi Dynasty and the establishment of the Islamic Republic, fostered a greater focus on justice and attention to underserved rural areas [6]. The primary health care (PHC) model, which had been piloted and developed in a region of Iran before the revolution, was scaled up and expanded nationwide in the early 1980s, providing a HSP across the country, particularly in rural areas [7].

From the beginning, the provision of services based on the needs of society, especially the vulnerable, has been a principal and was included in the HSP setting [8]. The same principle (coverage of needed services and financial protection) has been followed in forming a diagnostic and treatment services package. However, despite the significant achievements of Iran’s health system in increasing life expectancy and improving the population’s health [9, 10], the concern of low efficiency (high costs compared with outputs) has been seriously raised [11]. This issue has been exacerbated by the economic crisis caused by imposed economic sanctions and the COVID-19 pandemic in recent years [12] and seriously escalated the need to revise the HSP [13].

The history of measures taken to set HSP shows successes in generating local knowledge for prioritization, capacity building in related fields and establishing institutions to govern health technology assessment (HTA) and HSP setting. For example, the willingness to pay for gaining health [14] and social health insurance [15] were studied. In prioritization methodologies, the Technique for Order of Preference by Similarity to Ideal Solution (AHP-TOPSIS) approach was applied to some technologies [16]. In addition, several criteria were identified through experts’ opinions, including efficiency/effectiveness, safety, population size, vulnerable population size, availability of alternative technologies, cost-effectiveness, budget impact, financial protection and quality of evidence [17]. Another work assessed the public perspective regarding prioritization criteria and recognized disease severity, age, daily care needs, number of alternative interventions, individuals’ economic status and diseases with absence from work [18]. Despite all efforts, which some of them also are elaborated in the result section of the current paper, producing and using evidence to prioritize health services are not yet well embedded in Iran’s health system [19–21].

Lack of attention to taking effective initiatives to strengthen institutional capacities can hinder using evidence to develop HSP [22]. Institutionalization can be defined as the process by which a set of activities becomes an integral and sustainable part of a formal system. It can be seen as a sequence of events leading to ‘new practices becoming standard practice’ [23]. Institutionalization plays an important role in using evidence to support decisions that can help improve the health policy development processes and ultimately strengthen health systems [24]. In fact, due to the complex individualized, organizational and system relations of health organizations and the contextual circumstance [25], it is not easy to institutionalize using evidence to set and implement HSP [4]. Hence, the aims of this study are (1) to review the measures undertaken over the past four decades to establish the HSP in Iran and (2) to identify the obstacles encountered in institutionalizing evidence-informed priority setting for HSP. Notably, we have employed the Kuchenmüller et al. framework [23], which focuses on the Institutionalization of evidence-informed policymaking, to evaluate its applicability empirically for the first time.

## Methods

Study design: The study was qualitatively based on document analysis and semi-structured interviews with key stakeholders. We employed document analysis as a data collection method for systematic collection, documentation, analysis, interpretation and organization of printed or electronic data [26, 27] to mainly describe the actions taken to set the HSP and clarify the current situation in Iran. Additionally, we chose interviews to get an in-depth understanding of stakeholders’ various perceptions, particularly regarding the challenges of institutionalizing the evidence-informed prioritization efforts in Iran. We utilized the Standards for Reporting Qualitative Research (SRQR) checklist to present this study.

Reflexivity statement: In this study, all researchers are experienced in setting and implementing HSP within a low- and middle-income countries’ context. Among the researchers, one is female. Our professional backgrounds span health research policy and systems, epidemiology, health policy and financing. We collectively believe that health interventions funded by public resources should be set through a scientific and participatory approach, specifically a deliberative, evidence-informed process. While this approach is suitable for defining the HSP, its institutionalization and integration into the health system pose significant challenges and can be considered a crucial health reform necessary for moving toward UHC.

Study setting: Iran, with a population exceeding 85 million, was classified as a lower-middle-income country, at the time of study, in West Asia. Over the past decades, Iran has faced significant political events, including revolution, war and economic sanctions. Despite these shocks, the country has made notable progress in literacy, urbanization and investments in the transportation and food industries. In the health sector, various policies have been implemented across primary, secondary and tertiary care, as well as in medical education and research, to ensure ‘health for all’ [9]. While there have been improvements in health outcomes, several challenges persist, including the need for a sustainable financing mechanism to support effective health interventions.

Theoretical perspective: We found that the Kuchenmüller et al. [23] framework of institutionalizing evidence-informed health policymaking is useful as a practical lens to understand key stakeholders’ perspectives on key challenges for the successful institutionalization of evidence-informed prioritization. Based on this framework, there are six domains for a successful institutionalization, including: (1) governance; (2) standards and routinized processes; (3) partnership, collective action and support; (4) leadership and commitment; (5) resources; and (6) culture.

Document selection: Potentially, documents were identified through the following methods: (1) relevant government bodies and ministries website searches (e.g. Ministry of Health and Medical Education (MoHME), Ministry of Welfare, Parliament, Management and Planning Organization, Health Insurance Organizations), (2) Google search, (3) expert consultation with key informants and (4) hand searching in libraries.

Two authors (HSS and RM) reviewed the retrieved documents to validate the accuracy of their data and sources. To be eligible for inclusion, the documents had to meet the following criteria: (1) published after 1985 (the year of establishment of the MoHME); (2) relevant to HSP activities, including health need assessments, prioritization, pooling, fund allocation, budgeting, and health monitoring and evaluation; and (3) document types included books, papers, national development plans and policies, general health policies, bylaws, legal documents, technical reports and official guides. Documents were excluded if they did not specifically outline events and actions related to defining and implementing HSP or if a more recent version of the same document was available. Finally, 98 documents were included.

Data extraction: The details of included documents were entered into a data extraction matrix developed in Microsoft Excel, including the title, type, timeframe, main actions and events related to HSP development or implementation and the factors that have enabled or hindered this process. One author (HSS) extracted the data.

Study participants: We aimed to achieve diverse perspectives in our study. We employed a purposive sampling strategy, combining maximum variation sampling with a snowballing approach. It allowed us to identify potential participants with varying backgrounds in health policymaking, health financing, health sector reforms and health management. Given our relatively homogenous study populations and narrowly defined objectives [28], our sample consisted of nine individuals, ensuring data saturation. Documents and participants’ information is provided in Table 1.

**Table 1:** Characteristics of relevant documents and study participants

Document review			
Type of document	Code	Number	Name
Law	D1-24	24	Universal health insurance; national development plans; structure of the Welfare and Social Security system; structure and responsibilities of MoHME; enactments of the Cabinet; enactments of the Parliament
Policy	D25	1	General health policies
Regulation	D26-78	52	Enactments of the SCHI; approvals of health insurance
Instruction	D79-80	2	Health Transformation Plan; Family Physician Plan
Report	D81	1	Health Service Package development
Published books and papers	D82-89	18	–
In-depth Interviews			
Expertise	Code	Gender	Work experience (*y*)
Policymaking/planning	P1 P2	Male Female	More than 20 More than 20
Financing	F3 F4 F5	Male Male Male	More than 20 More than 20 More than 20
Service delivery	S6 S7 S8 S9	Male Female Male Male	10–20 10–20 More than 20 Less than 10

Interview procedure: We performed qualitative, in-depth interviews that lasted 30–50 min between February and July 2022. Two authors (HSS and RM) with experience in qualitative research conducted all interviews. We developed an interview guide with open questions for the semi-structured interviews. We slightly adapted the initial interview guide after conducting two interviews. The stakeholders were asked to explain what has been done in the last 40 years to define/revise the package in Iran, share their experiences about how these initiatives have strengthened the health system’s functions, what have been the impacts or consequences of these initiatives and express their ideas on the main challenges of institutionalizing the evidence-informed prioritization to set the HSP. Invitations, a consent form and study information were emailed. We used audio-recorded video or phone calls for the interviews and took field notes during the interviews. The audio files were then transcribed verbatim. Summaries of the interviews were emailed to stakeholders for revision and completion if necessary.

Data analysis: The same two authors who conducted the interviews also analysed the data using the framework analysis approach, guided by the Kuchenmüller et al. framework [23] as the analytical framework. The approach consists of seven stages: transcription, familiarization with the interviews, coding, development of a working analytical framework, application of the analytical framework, charting and data interpretation [29]. We imported the content from relevant documents and the transcripts of interviews into ATLAS-ti 8, a qualitative data analysis software. Subsequently, we immersed ourselves in the data by iteratively listening to the interview audiotape recordings and reading the interview transcripts and documents. During this immersion process, we annotated and coded key ideas and concepts. Our coding approach combined both inductive and deductive methods. We then organized the codes into categories and subcategories, which revealed the main challenges in institutionalizing evidence-informed priority setting. Through an iterative process, we developed a working analytical framework. This framework was applied to index subsequent transcripts and documents using the existing categories and subcategories. The resulting categories were charted into framework matrices to summarize the findings in a structured manner. Finally, we interpreted the data through discussions with all co-authors, resolving discrepancies through consensus.

The documents and interviews that were included were originally in Persian. The code descriptions and quotations, extracted from the interview transcripts and documents, were subsequently translated from Persian into English.

In our study, we employed Guba and Lincoln’s criteria to evaluate the trustworthiness of our work. These criteria are widely recognized in qualitative research and provide a robust framework for assessing the rigour and credibility of findings [30]. To this end, the credibility was established by prolonged engagement with participants and member checking. Besides, selecting participants with diverse experiences increases the possibility of shedding light on the research question from different aspects. Dependability and conformability were achieved through an auditing process. Two auditors examined the analytical process and the records of meetings for accuracy and then assessed whether all analytical techniques of the grounded theory had been used. The auditors reviewed the analysis of the descriptive, axial and selective codes to ensure whether they followed the study data. The research team documented all study data and described the participants and the research process to help assess the present findings’ transferability.

### Ethical consideration

In our study, we secured informed oral consent from all participants, emphasizing their voluntary participation without coercion. Before data collection, we transparently communicated the study’s purpose, procedures, potential risks and benefits. Participants were fully informed and had the opportunity to seek clarification or ask questions. To safeguard privacy, we rigorously maintained participant confidentiality. Identifying information was meticulously removed during data analysis and reporting. Our commitment to confidentiality extended to securely storing data, ensuring that only authorized researchers had access.

## Results

### Evolution of setting HSP in Iran

Iran’s HSP comprises two primary components, each with distinct characteristics and significant events shaping their development, including preventive and PHC Services and diagnostic and therapeutic Services.

Preventive and PHC services: At the first level of care, preventive and primary services are provided, focusing primarily on health promotion and prevention, with some surgical, pharmaceutical and diagnostic services included. Funded by the government, these services are freely accessible to the entire population [31, 32]. The private offices are the main providers of the first level of care, particularly in urban areas and populated cities where basic insurance schemes cover their services. The main policies and reforms that influenced the development and evolution of PHC services in Iran were as follows:

- 1970s – PHC pilot project: The initial phase involved a limited number of rural health houses. HSP focused on mothers and children, as well as environmental health and infectious disease services provided by community health workers. Target diseases were selected on the basis of hospital admissions and mortality causes, following WHO and UNICEF recommendations [33–35].
- 2000s – PHC scale-up: This phase included a comprehensive review of the HSP to expand service coverage. New services related to family and school health, noncommunicable diseases (NCDs), oral health, mental health and more were defined. Consideration was given to human resource needs and integrating different health system levels to maintain continuity of care through a referral system [33].
- 2005 − Family physician plan: Implemented in rural and nomadic areas, this plan introduced family physicians and established a referral system and rural health insurance [36, 37].
- 2015 – Updating PHC package of services: As part of Iran’s health reform aimed at achieving UHC, Health Transformation Plan (HTP) [38], the PHC services were revised to focus on specific population groups (children, adolescents, middle-aged, elderly and pregnant mothers). The revision included mental health, NCDs, nutrition, infectious diseases and environmental and occupational health services [39, 40].

Diagnostic and therapeutic services: This component encompasses diagnostic and para clinical services, clinical interventions, medicines, medical equipment and specialized care primarily provided in hospitals and ambulatory care centres. Significant initiatives in this area include:

- 1995 – Passive purchasing by basic insurance funds: The establishment of the Supreme Council of Health Insurance (SCHI) [31] marked the beginning of efforts to coordinate insurance organizations. One of the first major actions by SCHI was the publication of a negative list of non-covered services, including cosmetic surgeries, organ transplants and infertility treatments.
  “The beginning of the modern stage of the setting HSP in Iran goes back to 1994 when the issue of hospitals autonomy program was raised, and it was decided that the health insurance organization would be formed and the Universal Health Insurance Law was approved. In that law, it was the first place where we formally talked about the service package.” P4

“32 items have been excluded from the HSP under the title of additional and supplementary insurances, such as canes, walkers, and glasses.” P3

- 2007 – HTA: HTA was introduced to facilitate strategic purchasing. Several HTA projects have been conducted [41]. However, a review of completed HTA projects indicated that the application of HTA evidence was unsatisfactory, and HTA was not fully institutionalized in the decision-making process [21].
- 2015 – Strengthening strategic purchasing through Disease Control Priorities 3 (DCP3): During the monitoring of the HTP, several inefficiencies in the basic services insurance package were identified. Efforts were made to build capacity and move towards strengthening strategic purchasing to address these inefficiencies [10].
  “Lately, it was the same DCP3 approach that was started in departments such as maternal health, NCD, and AIDS, but it did not reach a result.” P8

The importance of DCP3 lies in its role as a global initiative advocating for the use of a prioritized list of cost-effective interventions, tailored for resource-constrained nations, endorsed by international organizations and promoted for use in these countries [4]. A country initiative was established to translate DCP3 for use in Iran. The initial assessment of this initiative indicated that DCP3 has limited added value in Iran, as the services provided in DCP3 are more appropriate for countries with limited service coverage.

- 2018 – Use of evidence-informed deliberative processes: The service package and medicines related to multiple sclerosis (MS) [42] and diabetes [43] were reviewed and endorsed by the SCHI in 2021 for implementation. In this process, three pillars were identified and used, which were: quality of care (effectiveness and safety), necessity (out of pocket payment and alternative availability) and sustainability (budget impact) [42].

### Challenges of institutionalizing evidence-informed prioritization to set HSP in Iran

After data analysis, six main categories emerged. According to participants’ statements, the barriers encompassed 23 subcategories (Table 2). In the following section, the most critical challenges of institutionalization are explained according to Iran’s experience in developing and revising the service package.

**Table 2:** Challenges of institutionalizing the evidence-informed prioritization in Iran to set and implement health service package

Category	Subcategory	Sample quote
Governance	Mandatory of revising the health service package based on an evidence based Weak legal framework Lack of well-established and harmonized structure	“Basically, the issue is that while the package revision is part of the national plans and policies, it's not really required. There’s no real push for it to be done with evidence.” P9 “We may have some solid rules in place for this package, but it’s all talk. There’s no actual infrastructure provided, and there’s no follow-up to see if the work is actually being done.” P1 “What we really need right now is a clear, step-by-step guide on how to develop and review the package with evidence, involving all stakeholders. We have the big picture, but the specifics are unclear.” P4 “When it comes to prioritizing our needs, we understand its importance, but we lack a system for it. For instance, we're unsure of where to find the relevant evidence to address these needs and how to effectively utilize it permanently.” P6 “Promoting decision-making and operation based on scientific evidence; and codifying standards and guidelines; undertaking health technology assessment; establishing the referral system by prioritizing prevention and health improvement and integrating them with the medical education system” D25
Standards and routinized processes	Lack of well-defined priority setting process Undefined criteria for priority setting Differences in priority setting in different departments Absence of a defined process for effective implementation of the health service package	“It has been almost a decade since the introducing of HTA in Iran, and if we take that as an example of using evidence in developing the package, we can see the impact it has had with numerous graduates and evidence produced, but we are not implementing HTA systematically, and if we had done so earlier, the status of the service package would be different.” P5 “The Ministry of Health has been closely managed HTA occasionally this progress, and the Food and Drug Organization has an office dedicated to this. for the same task different bodies with parallel tasks.” P2 “Do you remember when experts and specialists gathered for the DCP3? They all possess technical knowledge, but it still remains unclear who should do what and when. There are multiple organizations assigned to one task, while some tasks lack dedicated organizations.” P1 “However, there is still a lack of consensus among insurance providers regarding which interventions should be covered.” P6
Partnership, collective action and support	Conflicts of interest Managing interests is people’s preferences Diversity between licensing bodies for health technologies Insufficient collaboration within the organizations	“As mentioned before determining the type of intervention to include in the package is one of the most controversial issues.” P3 “The situation is quite complex. When prioritizing the well-being of society, it is crucial for the country and government to carefully consider how to navigate these conflicting objectives and make decisions that ultimately serve the best interests of the community.” P5 “The transfer of the Supreme Council of Insurance from the Ministry of Health to another ministry, along with its changing position over time, highlights a lack of constructive cooperation between organizations.” P7
Leadership and commitment	Lack of awareness among health managers Deficiency in necessary leadership skills Political considerations Management changes/turnover Managers’ value system	“The enthusiasm of top managers is key for this job. It's tough work that requires dedication and genuine interest. Dr. X had the chance to work on this project in his spare time, but the constant changes in management made it impossible to continue.” P4 “Special leadership skills, particularly in areas such as negotiation, advocacy, and motivation, are crucial for moving towards use of evidence. It is noted that this may be a weakness among senior managers.” P2 “In our country, the values held by decision-makers play a significant role in shaping the system. It is important to demonstrate the value of evidence-based decision-making to decision-makers, emphasizing its importance akin to principles like justice.” P8
Resources	Lack of multidisciplinary team working Insufficient financial resources Restrictions of information systems	“Similar to any system change, additional resources are required in the initial phases to thoroughly review the package based on evidence. While human resources may not be a primary concern at the moment, the availability of financial and informational resources is crucial.” P1 “We were indeed supposed to have dashboards and integrated health information systems. Currently, the availability and access to these tools may vary.” P9
Culture	Deficiency in accountability culture Lack belief in and reliance on evidence Failure to participatory approach Inadequate documentation practice	“It appears that progress in the DCP3 has been hindered by various factors, including cultural aspects.” P2 “While there is a belief in evidence-based practices, accountability and adherence to evidence may not be ingrained in the culture.” P6 “It may be necessary to implement measures to ensure compliance with evidence-based approaches, similar to enforcing seat belt usage in cars, to promote adherence to evidence-based practices.” P9

#### Governance

Our study revealed that the need to revise the HSP, particularly at the second and third levels of care, is not acknowledged as an essential component of the health system’s goal to achieve UHC. Participants highlighted that although revising the HSP through evidence-informed process has been incorporated into upstream policies, this approach has not been consistently implemented. It is largely attributed to the perception of such revisions as standalone endeavors rather than integral steps towards realizing UHC.

Participants further emphasized that this perception has led to the adoption of varying approaches in setting HSP across different levels of care, including inter-sectoral interventions, primary health care and secondary and tertiary care. It was also noted that these approaches do not form a cohesive package to promote health and ensure health equity for the entire society. It was emphasized that the current HSP lacks comprehensive addressing for implementing the general health policies (GHPs), per UHC principles.

The setting of these packages lack a systematic and integrated approach. There are instances of overlap or remnants of certain services, and the overall structure is unclear. The lack of a sustained and rigorous scientific approach to prioritizing health interventions results in limited access to essential and affordable services for vulnerable populations. It, coupled with substantial out-of-pocket expenses, undermines their financial protection.

Participants emphasized another obstacle which is the absence of a legal framework that endorses the use of evidence in prioritizing services. Nevertheless, sustainability of any advancements in this area would be challenging without sufficient enforcement and regulatory backing.

Another challenge identified is the lack of a well-defined organizational structure essential for deliberative evidence-informed prioritization. Participants noted that despite the establishment of structures such as the secretariat of the SCHI, the PHC Network Development Center and the HTA office, there are still deficiencies in the institutional arrangement. The roles, responsibilities and levels of authority within these units lack clarity. Furthermore, there are shortcomings in the composition of members, which impacts the involvement of relevant stakeholders and the definition of their roles.

#### Standards and routinized processes

Participants strongly believed that the main barrier to institutionalizing evidence in setting HSP was a lack of well-defined and clear processes. That is why there were different approaches for defining or revising the HSP. They described that most preventive and PHC services are recommendations of international agencies. In contrast, different criteria have been considered for assessing the eligibility of diagnostic and therapeutic services over time, including disease burden, effectiveness, safety and financial burden. They pointed out that decisions were made by consensus or voting, and criteria were not scored.

According to the participants, Iran has been setting HSP for decades, using different methodologies that are not usually clear. The process has traditionally relied on experts’ opinions, initially implicitly and explicitly, for the past 15 years. The criteria used have evolved in line with the changes and advancements in methodologies worldwide.

The study participants highlighted that apart from the inadequate process and criteria for prioritizing, there is also a notable absence of a defined process for effectively implementing the HSP. Additionally, the lack of a reliable mechanism for monitoring the outcomes of HSP implementation emerged as a significant challenge. Moreover, the participants expressed genuine concern about the inadequacy of a robust accountability mechanism to oversee the revision of the HSP, as endorsed in the country’s development plans and GHPs. Overall, these issues present considerable difficulties in providing quality and accessible services to the population, and policymakers must address these concerns and work towards strengthening governance and accountability mechanisms in the health sector to improve health outcomes for all.

#### Partnership, collective action and support

Regarding collaboration, participants highlighted a significant challenge in institutionalizing evidence uptake for setting HSP: conflicts of interest. The output of the HSP definition and revision processes influences the interests of diverse professional groups. However, it remains unclear why certain services are included in the HSP without clear engagement processes and mechanisms for stakeholders. Participants emphasized that the interests of powerful groups have manipulated the current HSP. One of the challenges lies in HTA offices outsourcing evidence production to the private sector and requesting them to fund studies. This practice increases the likelihood of conflicts of interest. Additionally, the diversity among licensing bodies for health technologies introduces complexities and may influence the final decision by interest groups.

In partnership, participants highlighted a significant challenge related to people’s preferences. From their perspective, there is no well-defined mechanism for actively listening to people’s voice. Some policymakers even question the eligibility of individuals to express their opinions. One participant pointed out that ignoring people’s voices and the existing conflict of interest has led to people increasingly valuing services provided by specialists. As a result, specialization has come to dominate the service-seeking behaviour.

Lack of collaboration within the organizations involved in setting HSP was a significant barrier to the institutionalization of using evidence in priority-setting. One of the key observations from experts in this field is that many organizations operate in silos and do not communicate effectively with each other. This lack of collaboration leads to duplication of efforts, conflicting priorities and the generation of unreliable or incomplete data. Participants specified that the absence of a culture of collaboration makes it difficult for organizations to share information, knowledge and resources necessary for producing high-quality evidence. As a result, using evidence in decision-making becomes ad hoc and less effective. The institutionalization of using evidence requires a culture of collaboration among stakeholders, including policymakers, researchers, health professionals and community members.

#### Leadership and commitment

The participants identified a significant challenge to effectively institutionalizing the prioritizing HSP: a lack of awareness among health managers and senior leaders about the benefits of evidence-informed methods. According to the participants, many of these managers are clinical service providers with limited knowledge of management science and economics. As a result, they tend to rely on traditional methods of prioritizing services based on their clinical expertise rather than incorporating evidence-informed approaches. This shortage of awareness and commitment among managers hinders the sustainability of change efforts and perpetuates the use of less effective, outdated methods.

The participants also identified a lack of necessary leadership skills as another obstacle to effectively institutionalizing use of evidence-informed approaches for HSP. Institutionalizing this change in the health system requires strong, charismatic leadership to guide, motivate and provide ongoing support. The shift from traditional methods is a fundamental change, and Iran’s successful experience in implementing PHC highlights the potential for another significant shift with powerful leadership. However, the participants noted that such leadership has been scarce in recent years, which is unfortunate for the health system and the people it serves.

The participants identified political considerations as another obstacle. In a such political environment, policymakers showcase their achievements to the public during election. However, since the benefits of evidence-informed approaches may not be immediately visible to the public and sometimes it results conflict with the interest of certain groups, policymakers may be hesitant to challenge themselves to prioritize it.

Some participants emphasized the challenge of management changes. Specifically, they shared their experiences of encountering managers who supported using evidence only to have them suddenly replaced for political reasons. These changes often occur without regard for technical knowledge or expertise, which makes it challenging to sustain an evidence-informed approach to service prioritization. Frequent management changes cultivate confusion, uncertainty and inconsistency within the organization, interfering with the successful implementation and institutionalization of evidence-informed strategies.

The managers’ and key decision-makers value systems were another significant obstacle reported by participants. From their perspective, most decision-makers do not believe the benefits of evidence-informed approaches, rely more on intuition and established habits when making decisions. This reluctance to embrace evidence-informed practices is a common issue that has been observed in various health systems across the world.

#### Resources

Participants highlighted that the most significant obstacle to institutionalizing the use of evidence in developing HSP was related to financial resources. They acknowledged that dedicated and sustainable financial resources are key to (1) implementing HSP and (2) establishing evidence-informed priority setting. Unfortunately, despite increased resources within the health sector, participants found that this approach was not prioritized in the actual allocation of financial resources. They added that allocating financial resources for short-term projects was insufficient and could not support the institutionalization of evidence-informed priority setting. Without a sustainable financial plan, integrating and maintaining an evidence-informed approach is challenging.

According to many participants, there has been a significant investment in capacity building in Iran to produce and utilize evidence for health policies. From their perspective, the quantity and quality of these resources are sufficient to institutionalize the use of evidence-informed practices. However, the cultural aspect remains an obstacle in terms of the lack of the necessary skills to work in multidisciplinary groups.

Participants acknowledged the importance of human resources from a wide range of fields, including economics, management, politics, social sciences and methodological sciences, to effectively institutionalize the use of evidence in different stages of policy development. However, it appears that the formation and activity of these groups are still in the early stages.

Participants recognized that Iran’s health information systems have improved. However, they indicated that the registration systems, routine data systems and data from national studies are not integrated enough to provide the necessary data for developing or revising the packages. Furthermore, these systems lack the capability to demonstrate changes in health system indicators resulting from implementing these packages, making monitoring and evaluating their implementation challenging. In other words, there is a gap in the health information system’s capacity to provide data relevant to evaluating the impact of evidence-informed practices in health policies.

#### Culture

The lack of an accountability culture was identified as a significant cultural barrier to using evidence in health policymaking. Participants noted that health managers have not been held accountable for not incorporating evidence in HSP development, and there is no mechanism to track the use or non-use of evidence. In addition, many managers do not view evidence as a critical component in developing HSP, which diminishes the motivation to adopt evidence-informed practices. This lack of accountability is not only limited to managers as users of evidence but also includes producers of evidence. Participants observed a lack of motivation among evidence producers to generate essential evidence required for package formulation due to the deliberative nature of the process.

As observed in the ‘Leadership and commitment’ section, another significant cultural barrier to using evidence in policy formulation is the lack of belief and habit of utilizing evidence. Participants noted that, in society, there is a greater emphasis on solving problems in the shortest time rather than obtaining and utilizing evidence to inform decisions. This approach overlooks the importance of evidence in improving health outcomes and undermines the value of evidence-informed practices. Moreover, as there is no accountability system in place, there is little concern for the impact of these decisions on the people affected.

## Discussion

Setting and implementing HSP, drawing upon both global and local evidence, exemplifies the use of evidence in policymaking. Beyond mere evidence utilization, HSP development necessitates active stakeholder engagement and alignment with local values to yield meaningful outcomes. However, pursuing UHC through HSPs is a gradual, long-term endeavour that does not yield immediate results [44].

The finding demonstrated that several efforts have been made to perpetuate evidence-informed prioritization process in health systems, starting in the 1970s and continuing in the 2000s, 2015 and 2019. However, they have yet to be institutionalized. Without robust institutionalization, the decision-making process underlying HSP prioritization may remain unstable and potentially ineffective, resulting in a wish list of services which does not have any real world implication for the countries. Therefore, a critical factor emphasized in setting and implementing HSP is institutionalization in achieving programmatic goals and advancing towards UHC. Furthermore, experience underscores those countries facing constant political instability or shock conditions need resilient decision-making processes, which can only be achieved through effective institutionalization [4, 5].

Institutionalization is a complex endeavour that necessitates careful consideration of contextual strengths and weaknesses to identify optimal solutions for addressing critical areas. Our study identified several key challenges in this process, including legal gaps, methodological diversity, fragile partnerships, leadership transitions, insufficient financial support and a culture of lack of accountability. While addressing all these challenges remains crucial, we contend that the primary focus areas should involve establishing transparent and accountable mechanisms and fostering public participation to strengthen partnerships, enhance collaboration and amplify the voices of vulnerable populations.

Moreover, it is imperative to establish a robust legal framework and a transparent process aligned with it to ensure comprehensive implementation. Realizing these measures demands high political commitment, persistent effort and effective mobilization of enduring resources to implement the results defined by the HSP.

As our findings highlighted, one of the significant challenges in the design and implementation of the HSP is the conflict of interest. To address this issue, it is imperative to establish or strengthen transparency and accountability processes. Recent service package review activities for MS and diabetes have emphasized transparency [42, 43], which is crucial for the success of these reviews and has been a persistent challenge in the recent HTA in the country [21]. A study in Iran highlighted the necessity of establishing accountability mechanisms to ensure policymakers make informed decisions [45]. Without these mechanisms to monitor actions and enforce appropriate rewards or penalties, the benefits of evidence-based prioritization cannot be realized. Implementing policies and decisions related to the HSP based on rigorous studies and expert opinions while reducing political influence, especially for costly technologies and medicines, are additional measures to enhance transparency and accountability.

Another notable challenge lies in disregarding the perspectives of vulnerable communities. Reviews of the HSP frequently show that public participation has either been neglected or considered superficially. Moreover, the principle of including vulnerable populations in developing the HSP has been ignored in all aspects of HSP [3]. Therefore, it is crucial to establish a platform for the participation of disadvantaged groups, allowing their voices to be heard. This approach incorporates the concept of intersectionality in public participation to institutionalize the HSP [46]. Especially in times of shocks and crises, when vulnerable groups are the first to be affected, it is crucial to consider intersectionality with a systemic approach [47]. In Iran, legal frameworks for public participation in policymaking exist but have largely been overlooked by policymakers. Public participation structures in Iran are defined in two forms: the health participation house and the health assembly. The health participation house serves as a broker between people and managers, facilitating communication and collaboration, while the health assembly involves the public directly in policymaking processes [48]. These structures can be leveraged to institutionalize public participation or similar mechanisms in the evidence-informed deliberative prioritization process of the HSP.

Beyond transparency and accountability, two other pivotal dimensions of institutionalization involve establishing a legal framework for evidence-informed prioritization and consolidating processes. It ensures a well-defined approach to priority-setting that incorporates empirical data and actively engages relevant stakeholders. In this context, revising the HSP is perceived as an integral part of achieving UHC. Without such deliberate efforts, sporadic interventions remain isolated and cannot effectively contribute to the defined goals. Drawing from the Iranian experience, an initial focus on specific disease groups or programs can mitigate sensitivities, promote sustainability and pave the way for broader optimization. For example, optimizing MS services ensures efficient resource utilization, preventing patient and provider disruptions. However, a comprehensive revision of the HSP remains essential for achieving UHC and enhancing overall health system efficiency [49]. Therefore, it should foster commitment and motivation among policymakers and ministries, emphasizing that evidence-informed decisions for public health funding are essential for ensuring health for all.

The prerequisites for institutionalizing evidence use in HSP become feasible when strong leadership and ample resources are in place. High levels of political commitment and sustained effort are essential for mobilizing the necessary resources and supporting the continuous development and implementation of the HSP [4]. Achieving this requires a fundamental shift in policymakers’ attitudes to ensure sustainable political backing for evidence-informed decision-making processes related to service packages. Without this transformation, persistent health system challenges—financing and service delivery—will continue exacerbating, hindering progress towards UHC [5].

### Strengths and limitations

In terms of methodology, we employed triangulation through document review and interview methods. Based on the convergence of responses, it can be asserted that the study demonstrates a suitable level of rigour. We used the framework proposed by Kuchenmüller et al. for data gathering and analysis. Empirical data validated this framework. While the framework proves helpful, it is essential to acknowledge its limitations. Notably, the framework lacks clear boundaries in critical areas such as governance and partnership, collective action and support, suggesting areas for improvement. Considering the findings, the significance of the work extends beyond optimizing HSP institutionalization and moving towards UHC. It also holds broader potential for institutionalizing evidence-informed policymaking.

However, our work has a notable limitation: we did not specifically address how the institutionalization of evidence-informed approaches may differ in countries experiencing conflict. The turbulent economic, social and political circumstances resulting from conflicts pose significant challenges to effective evidence-informed prioritization in health. Therefore, there is a call for more targeted investigations to implement effective and equitable health priority settings tailored to the unique contexts of conflict-affected countries.

## Conclusions

In general, it can be learned that in the context of HSP, what is referred to as technical cooperation should strongly emphasize sustainability. Defining the HSP and its implementation, which involves changes in resource allocation methods and power dynamics, is indeed a political-economic issue. Merely addressing it as a strategic purchasing topic or conducting a comprehensive economic evaluation falls short. Attention must be paid to the readiness for change in countries, and thorough preparations for implementation are essential.

A strategic approach is necessary to foster linkage and exchange between policymakers and technical assistance providers. It includes seizing windows of opportunity for policy change. Additionally, capacity-building efforts should focus on strengthening leadership skills and avoiding conflicts of interest. Institutionalizing service prioritization and evidence-informed policymaking demands attention to various dimensions.

International organizations and academic institutions from high-income countries, if aiming to assist low and middle-income countries in the field of HSP, should prioritize institutional strengthening. Technical cooperation alone will not ensure sustainability unless it achieves the necessary institutionalization. Therefore, guidance and capacity-building initiatives are essential for successful institutionalization alongside the existing framework.

## Data Availability

The data that support the findings of this study are available from the corresponding author, [RM], upon reasonable request.

## Abbreviations

AHP-TOPSIS: Analytic Hierarchy Process Technique for Order of Preference by Similarity to Ideal Solution
DCP: Disease Control Priorities
GHPs: General Health Policies
HSP: Health Service Package
HTA: Health Technology Assessment
HTP: Health Transformation Plan
MoHME: Ministry of Health and Medical Education
MS: Multiple Sclerosis
NCDs: Non-Communicable Diseases
PHC: Primary Health Care
SCHI: Supreme Council of Health Insurance
SRQR: Standards for Reporting Qualitative Research
UHC: Universal Health Coverage
UNICEF: United Nations Children’s Fund
WHO: World Health Organization
